# Multivariate epidemiologic analysis of type 2 diabetes mellitus risks in the Lebanese population

**DOI:** 10.1186/1758-5996-6-89

**Published:** 2014-08-21

**Authors:** Michella Ghassibe-Sabbagh, Mary Deeb, Angelique K Salloum, Francis Mouzaya, Marc Haber, Yasser Al-Sarraj, Youssef Chami, Yasmine Akle, Kamal Hirbli, Rita Nemr, Rechdi Ahdab, Daniel E Platt, Antoine B Abchee, Hatem El-Shanti, Pierre A Zalloua

**Affiliations:** School of Medicine, Lebanese American University, Beirut, 1102 2801 Lebanon; Shafallah Medical Genetics Center, Doha, Qatar; Centre Hospitalier du Nord-CHN, Zgharta, Lebanon; Bioinformatics and Pattern Discovery, IBM T. J. Watson Research Centre, Yorktown Hgts, NY 10598 USA; Department of Internal Medicine, American University of Beirut, Beirut, Lebanon; Harvard School of Public Health, Boston, MA 02215 USA

**Keywords:** Type 2 diabetes, Prediabetes, Lebanon, Risk factors, Hyperlipidemia, Coronary artery disease

## Abstract

**Background:**

The burden of diabetes in Lebanon requires well-targeted interventions for screening type 2 diabetes mellitus (T2DM) and prediabetes and prevention of risk factors. Newly recruited 998 Lebanese individuals, in addition to 7,292 already available, were studied to investigate the prevalence of diabetes, prediabetes and their associated risk factors.

**Methods:**

Participants had fasting blood sugar and glycohemoglobin tests in addition to a lipid profile. Clinical and demographic information were obtained from a detailed questionnaire. The relationship between T2DM, its risk factors, and its complications were tested. Comparisons of these risk factors among diabetics, healthy, and coronary artery disease (CAD) patients were performed.

**Results:**

The prevalence of T2DM significantly increased with increasing BMI (p < 0.0001). Exercise activity level negatively correlated with the disease (p = 0.002), whereas the prevalence of T2DM (p < 0.0001) and CAD family history (p = 0.006) positively correlated with the affection status. The mean levels of triglycerides and LDL-C were significantly higher in diabetics (1.87; 1.35) compared to individuals with prediabetes (1.63; 1.26) and unaffected controls (1.49; 1.19). People with T2DM showed a significant decrease in HDL-C levels. A strong correlation of overall hyperlipidemia with the diabetes affection status was shown (p < 0.0001). Other comorbid factors such as hypertension (p < 0.0001) and self-reported obesity (p < 0.0001) were highly associated with T2DM and prediabetes. Reproductive health of women showed a strong correlation between giving birth to a baby with a high weight and the occurrence of T2DM and prediabetes later in life (p < 0.0001). Retinopathy and peripheral neuropathy were significantly correlated with diabetes and prediabetes (p < 0.0001).

**Conclusions:**

The present study shows an alarming prevalence of diabetes and prediabetes in the studied subgroups representative of the Lebanese population.

**Electronic supplementary material:**

The online version of this article (doi:10.1186/1758-5996-6-89) contains supplementary material, which is available to authorized users.

## Background

According to the International Diabetes Federation (IDF), 32.8 million people or 9.1% of the adult population in the Middle East and North Africa Region (MENA) had diabetes in 2011 [1]. A further 24 million people, or 6.7% of the population, are at high risk of diabetes from impaired glucose tolerance. Six of the 10 countries with the highest prevalence of diabetes in the world are from the MENA Region: United Arab Emirates (18.7%), Saudi Arabia (16.8%), Bahrain (15.4%), Kuwait (14.6%), Oman (13.4%), and Lebanon (7.7%). By 2025, the number of people with diabetes is expected to more than double in these regions. According to the World Health Organization, the age-standardized mortality rate from cardiovascular disease and diabetes per year was estimated to be 224 in 2008 in Lebanon, which is 1.2 times higher than cancer mortality rate.

In spite of the high estimates of diabetes prevalence in the region, the healthcare expenditures due to diabetes account for 2.3% of the total global figure and many governments remain largely unaware of the current magnitude and future burden of diabetes in their societies [1]. T2DM in Lebanon would be better managed if we had a global overview on the prevalence of prediabetes and diagnosed and undiagnosed diabetes mellitus, coupled to an estimation of risk factors such as physical inactivity, obesity, hyperlipidemia, and gestational diabetes mellitus (GDM). In addition, T2DM and Coronary Artery Disease (CAD) frequently coexist and are major components of the public health and economic burden worldwide. An epidemiologic study targeting risk factors such as physical inactivity, smoking, and alcohol consumption can therefore help guiding a proportionate public health response to the epidemic of diabetes and CAD. Susceptibility to these complex diseases is strongly influenced by common environmental factors, but how these factors act in the Lebanese population remains largely unknown.

In order to leverage the genetic information from prior studies in this laboratory, it is desirable to pool prior CAD data with data collected specifically for T2DM. A difficulty is that the enrollment criteria were strikingly different. The CAD study subjects were all enrolled from a pool of catheterized patients. Since catheterization is an invasive procedure, all subjects, whether or not they ultimately showed luminal narrowing, had to showsufficient cause for the catheterization according to clinical indications and behavior. The T2DM study enrollment occurred at multiple clinical sites where recruitment was advertised. This enrollment included selection of cases versus controls. In order to associate genetic markers with demographics and disease in a pooled set of data, the impact of the enrollment criteria was characterized.

The aims of this study are: (1) to estimate the prevalence of prediabetes, diagnosed and undiagnosed diabetes in the Lebanese population age 55 or older, (2) to investigate and identify the risk factors of T2DM and CAD in the Lebanese population and, (3) to estimate the inferred risk of known factors to T2DM and CAD patients. Results from this study will help policy makers and health practitioners in promoting well-targeted prevention programs to minimize the burden of diabetes mellitus in developing countries.

## Methods

### Study population

The study consisted of two phases. In the first phase of the project we actively recruited 998 subjects in 2012 specifically to survey the prevalence of diabetes and its risk factors among the Lebanese population. In the second phase we selected 991 CAD cases from an existing database that consisted of 7,292 subjects recruited between 2007 and 2013 to study CAD and associated diseases such as T2DM and hypertension (FGENTCARD - Functional Genomic Diagnostic Tools for Coronary Artery Disease). The combined phase 1 and phase 2 datasets were used to study the association of risk factors with T2DM and CAD and to estimate the inferred risk of known factors to T2DM and CAD.

An additional CAD-free 1,330 subjects from the FGENTCARD database were combined with the subjects from Phase 1 to further investigate the prevalence of T2DM in the Lebanese population.

#### Surveyed population through targeted T2DM campaigns (Phase 1)

In this phase 998 Lebanese adult subjects were recruited through two targeted advertisement campaigns for volunteers over the age of 55 to have their blood tested for diabetes. The first campaign was conducted in the suburbs of Beirut, the capital of Lebanon, with the collaboration of the Lebanese American University Medical Center. The second advertisement campaign was conducted in North Lebanon where volunteer recruitment was done in five villages with the help of endocrinologists and local social workers. Research was carried out in compliance with the Helsinki Declaration and with the approval of the LAU Institutional Review Board and local ethics committees on human research (Reference number SMPZ08072010-4). All participants signed an approved informed consent. The consent form stipulated that the participant agreed to be interviewed and to give a blood sample to be used for DNA analysis, as well as glycated hemoglobin (HbA1C), fasting blood sugar (FBS), and lipid profile measurements.

#### FGENTCARD population (Phase 2)

991 subjects, of which 31.9% were diabetics and 68.1% were non diabetics were selected from a database that included 7,292 subjects that were recruited between 2007 and 2013 for inclusion in the FGENTCARD database. Detailed clinical and demographic information have been previously described [2] (Additional file 1). 406 out of the 991 participants had >50% stenosis in at least one vessel and were designated as severe CAD patients, 207 had stenosis ≤50% in at least one vessel and were designated as moderate CAD patients, and 378 had 0% stenosis and were classified as controls. Individuals were chosen to match with the T2DM surveyed populations for sex (35.6% of males and 64.4% of females) and mean age (63.9 for severe stenosis; 65.9 for moderate and mild stenosis and 63.7 for unaffected controls).

The subjects from Phase 1 were compared to a stenosis-free population of 1,330 clinically well-characterized subjects selected from the FGENTCARD database. From the 1,330 selected controls, 664 were more than 55 years old.

### Blood samples collection and laboratory test performance

In order to measure the height and weight of all participants, a calibrated mass was placed on a scale. The known mass value was compared to the scale reading and the percent error was calculated to determine the acceptability of the balance or scale prior to height and weight measurements. The height and weight were used to calculate the body mass index (BMI). The blood samples were collected by well-trained phlebotomists.

For the surveyed population, blood samples were collected for Glycated Hemoglobin 1C (HbA1C) testing, FBS and lipid profile assays. HbA1C, Fasting Blood Sugar (FBS) and lipid profile assays were performed on COBAS INTEGRA 400 Plus as follows: HbA1c levels were determined using an immunoturbidimetric assay, FBS was measured via an enzymatic reference method using the hexokinase, and the lipid panel (total cholesterol (TC), triglycerides, HDL-cholesterol (HDL-C), and LDL-cholesterol (LDL-C)) was measured according to the absorbance photometry principle via enzymatic colorimetric methods.

For the FGENTCARD population, cardiologists performing the coronary angiography collected a 20 mL blood sample from the peripheral femoral artery of patients. Annotations were coded from medical charts according to the study protocol, which included results of laboratory tests, prescribed medications, and presence of other diseases and conditions.

### Measurement of variables

BMI was calculated according to standard measurements. A BMI of 25–30 kg/m^2^ indicated overweight and a BMI of ≥30 kg/m^2^ was an indicator of obesity. FBS was categorized according to glucose level: 70–99 mg/dL, normal fasting glucose; 100–125 mg/dL, impaired fasting glucose or prediabetes; and ≥126 mg/dL, diabetes. An HDL-C level <35 mg/dL was set as an increased risk of T2DM. For LDL-C, TC, and triglycerides, the threshold levels for an increased T2DM risk were ≥130 mg/dL, ≥200 mg/dL, and ≥250 mg/dL respectively in line with standard definitions of hyperlipidemia.

Participants were considered hypertensive if blood pressure is at or above 140/90 mmHg or if they were on antihypertensive drugs. Participants were diagnosed with T2DM if they were clinically documented diabetic patients on medication (oral hypoglycemic agent or insulin), or presented with a level of HbA1C of ≥6.5% in line with the American Diabetes Association (ADA) definition of type 2 diabetes mellitus diagnosis [3]. Individuals with an HbA1C ranging from 5.7% to 6.4% were considered as prediabetics with an increased risk of developing diabetes in the future. Individuals with an HbA1c ≤5.6%. were labeled as healthy non-diabetic. Participants were considered hyperlipidemic if they had clinically documented hyperlipidemia with medication intake, or presented with a level of TC, triglycerides, or LDL-C above the threshold. A family history was positive when the condition was present in a parent, sibling, or second degree relative.

### Statistical analysis

Data analysis was conducted with the statistical software package "Statistical Package for Social Sciences" (SPSS) for windows, version 20, and the glm package from R version 3.0.2 [4]. Chi square test is used to assess the association between two categorical variables and ANOVA for continuous variables. Odds ratios were obtained using multinomial logistic regression. For HDL-C, odds ratios were calculated using the thresholds of HDL-C <35 mg/dL and of TC/HDL-C ratio ≥4 mg/dL as increased risks for T2DM and CAD respectively. A *p* value of 0.05 was considered to indicate statistical significance.

Impact of enrollment was estimated by applying logistic regression to predict diagnosis of CAD and T2DM based solely on study population, where the data were re-partitioned for this analysis (1 = CAD study enrollment, 5,359 subjects; 2 = T2DM enrollment, 998 subjects, all of both groups 55 years old or older). Only 177 T2DM enrolled subjects showed knowledge of CAD state. Enrollment criteria associations were also explored for behavioral components (active lifestyle and smoking), as well as clinical variables (diagnoses of hypertension, hyperlipidemia, high triglycerides, BMI – threshold of 30, and HDL). Second, association of enrollment population and behavior with clinical variables was assessed. Third, an association test of how metabolic syndrome varies with diagnosis of T2DM and CAD was performed. Fourth, interactions of triglycerides with T2DM, and the impact of T2DM on CAD, were examined. These results are non-tabulated due to variable format and test structure.

## Results

In the surveyed population (Phase 1) 354 (35.6%) were males and 639 (64.4%) were females (Table 1). The mean age of the control group is 65 years old which diminishes the possibility of developing T2DM since the mean age of onset of T2DM in our cohort was 54.6 years old (±12.4). The prevalence of T2DM significantly increased with increasing BMI (p < 0.0001) (Table 1).

**Table 1:** **Distribution in the surveyed population of different factors by T2DM diagnosis status**

	T2DM categories				
	Diabetics n = 407	Prediabetics n = 207	Non-diabetic n = 379	Total n = 993	***p***value
	No. (%)	No. (%)	No. (%)		
**Gender**					**0.4**
Male	154 (37.8)	68 (32.9)	132 (34.8)		
Female	253 (62.2)	139 (67.1)	247 (65.2)		
**Age (Years) mean (SD)**	64 (11.4)	66 (9.9)	65 (12.3)		**0.09**
**Age of onset (Years) mean (SD)**	55 (12.4)	60 (11.4)	NA		**0.03**
**Body Mass Index, mean (SD)**	29.1 (5.4)	28.6 (5.1)	27.3 (4.4)		**<0.0001**
**Exercise activity level**					**0.9**
Non-active	248 (62.0)	116 (56.9)	215 (60.4)		
Moderate activity	58 (14.5)	33 (16.2)	48 (13.5)		
Heavy activity	94 (23.5)	55 (27.0)	93 (26.1)		
**Smoking**					**0.2**
Never smoker	179 (44.8)	103 (50.5)	190 (53.2)		
Former smoker	92 (23.0)	43 (21.1)	70 (19.6)		
Current smoker	129 (32.3)	58 (28.4)	97 (27.2)		
**Alcohol consumption**					**0.003**
Never drinker	276 (69.0)	132 (64.7)	219 (61.3)		
Less than 7 drinks per week	118 (29.5)	70 (34.3)	119 (33.3)		
More than 7 drinks per week	6 (1.5)	2 (1.0)	19 (5.3)		
**Family history of T2DM**					
Yes	267 (67.9)	87 (44.4)	158 (47.7)		**<0.0001**
No	126 (32.1)	109 (55.6)	173 (52.3)		
**Consanguinity**					**0.96**
Yes	69 (17.5)	36 (18.3)	58 (17.4)		
No	325 (82.5)	161 (81.7)	276 (82.6)		

*Abbreviation*: *T2DM* Type 2 diabetes mellitus, *SD* Standard deviation.

Out of the 991 subjects selected in phase 2, 353 (35.6%) were males and 638 (64.4%) were females (Table 2). The mean age of the control group is 64 years old (±10.5) (n = 378) compared to 66 years old (±9.9) in the moderately affected group (n = 207), and 64 years old (±11.3) in the severely affected group (n = 406). Exercise activity level negatively correlated with the disease (p = 0.002), whereas the prevalence of T2DM (p < 0.0001) and CAD family history (p = 0.006) positively correlated with the affection status (Table 2).

**Table 2:** **Distribution in 991 individuals from FGENTCARD of different factors by CAD diagnosis status**

	CAD categories				
	Severe CAD n = 406	Mild CAD n = 207	Healthy n = 378	Total n = 991	***p***value
	No. (%)	No. (%)	No. (%)		
**Gender**					**0.5**
Male	153 (37.7)	68 (32.9)	132 (34.9)		
Female	253 (62.3)	139 (67.1)	246 (65.1)		
**Age (years) mean (SD)**	64 (11.3)	66 (9.9)	64 (10.5)		**0.04**
**Body mass index, mean (SD)**	29.1 (5.3)	29.7 (5.9)	29.7 (5.8)		**0.2**
**Exercise activity level**					**0.002**
Non-active	85 (21.0)	34 (16.5)	49 (13.1)		
Moderate activity	315 (77.8)	163 (79.1)	307 (81.9)		
Heavy activity	5 (1.2)	9 (4.4)	19 (5.1)		
**Smoking**					**0.01**
Never smoker	150 (37.0)	103 (49.8)	185 (49.1)		
Former smoker	105 (25.9)	43 (20.8)	81 (21.5)		
Current smoker	150 (37.0)	61 (29.5)	114 (29.4)		
**Family history of CAD**					
Yes	260 (64.5)	91 (44.2)	174 (46.3)		**0.006**
No	143 (35.5)	115 (55.8)	202 (53.7)		
**Consanguinity**					**0.44**
Yes	73 (19.6)	34 (17.2)	60 (16.7)		
No	299 (80.3)	163 (82.7)	299 (83.3)		
**Hypertension**					**0.1**
Yes	263 (64.8)	137 (66.2)	220 (58.5)		
No	143 (35.2)	70 (33.8)	156 (41.5)		
**Hyperlipidemia**					**<0.0001**
Yes	227 (56.0)	91 (44.0)	149 (39.5)		
No	178 (44.0)	116 (56.0)	228 (60.5)		
**Type 2 diabetes mellitus**					**<0.0001**
Yes	172 (42.4)	59 (28.5)	84 (22.3)		
No	234 (57.6)	148 (71.5)	292 (77.7)		

*Abbreviation*: *CAD* Coronary artery disease, *SD* Standard deviation.

To counter the recruitment bias in our selection criteria through the two T2DM advertised campaigns where it is expected that more diabetics would likely participate, we selected an additional 1,330 subjects stenosis-free from the FGENTCARD population. Out of these 687 (51.7%) were males and 643 (48.3%) were females (Table 3). The mean age of the control group is 55 years old (±11.9) compared to the mean age of the diabetics which is 58 years old (±11.6). According to their medical records, 20.5% of the participants had type 2 diabetes, this percentage increased to 23.6% when the calculations included people above 55 years old (Table 4). Studying risk factors by T2DM diagnostic status confirmed a significant association of activity level, BMI, triglycerides, overall hyperlipidemia, positive T2DM family history, and hypertension with T2DM (Table 3). Although HDL-C did not show significant association, there was a trend towards association of low levels of HDL-C and T2DM occurrence (Table 3).

**Table 3:** **Distribution in 1,330 random FGENTCARD individuals of different factors by T2DM diagnosis status**

	T2DM categories			
	Diabetic n = 272	Non-diabetic n = 1,058	Total n = 1,330	***p***value
	No. (%)	No. (%)		
**Gender**				**0.008**
Male	121 (44.5)	566 (53.5)		
Female	151 (55.5)	492 (46.5)		
**Age (years) mean (SD)**	58 (11.6)	55 (11.9)		**0.0002**
**Body mass index**				**<0.0001**
Less than 30	111 (42.2)	645 (62.6)		
More or equal to 30	152 (57.8)	385 (37.4)		
**Exercise activity level**				**0.007**
Non-active	41 (15.1)	93 (8.8)		
Moderate activity	214 (79)	886 (83.7)		
Heavy activity	16 (5.9)	79 (7.5)		
**Smoking**				**0.001**
Never smoker	143 (52.6)	433 (40.9)		
Former smoker	52 (19.1)	200 (18.9)		
Current smoker	77 (28.3)	425 (40.2)		
**Family history of T2DM**				
Yes	190 (69.9)	492 (46.5)		**<0.0001**
No	82 (30.1)	565 (53.5)		
**Consanguinity**				**0.6**
Yes	56 (21.2)	201 (19.7)		
No	208 (78.8)	818 (80.3)		
**Triglycerides**				
High level	162 (68.4)	505 (54.9)		**0.0002**
Normal level	75 (31.6)	415 (45.1)		
**HDL-cholesterol**				**0.6**
Low level	73 (30.7)	267 (29.0)		
Normal level	165 (69.3)	654 (71.0)		
**Hypertension**				**<0.0001**
Yes	197 (72.4)	487 (46.0)		
No	75 (27.6)	571 (54.0)		
**Hyperlipidemia**				**<0.0001**
Yes	213 (78.6)	659 (62.3)		
No	58 (21.4)	398 (37.7)		
**Family history of CAD**				**0.3**
Yes	144 (53.1)	602 (57.0)		
No	127 (46.9)	455 (43.0)		

*Abbreviation*: *T2DM* Type 2 diabetes mellitus, *CAD* Coronary artery disease; *SD* Standard deviation.

**Table 4:** **Distribution of the different populations studied by T2DM diagnosis status**

	Diabetic (%)	Non-diabetic (%)	Total
**Surveyed T2DM population**	407 (41)	586 (59)	993
**FGENTCARD population**	2,437 (33.4)	4,855 (66.5)	7,292
**Randomly selected population**	272 (20.5)	1,058 (79.5)	1,330
Randomly selected population (age ≥ 55 years old)	157 (23.6)	507 (76.4)	664
**CAD population**	2,152 (36.5)	3,748 (63.5)	5,900

*Abbreviation*: *T2DM* Type 2 diabetes mellitus, *CAD* Coronary artery disease.

In the surveyed population (Phase 2) the mean levels of triglycerides and LDL-C were significantly higher in diabetics (1.87; 1.35) compared to individuals with prediabetes (1.63; 1.26) and unaffected controls (1.49; 1.19) (Table 5). People with T2DM showed a significant decrease in HDL-C levels (Table 5). A strong correlation of overall hyperlipidemia with the diabetes affection status was shown (p < 0.0001) (Table 6). Other comorbid factors such as hypertension (p < 0.0001) and self-reported obesity (p < 0.0001) were highly associated with T2DM and prediabetes (Table 6). Although cardiovascular disease status did not show significant results, there was a positive trend (p = 0.08) (Table 6).

**Table 5:** **Lipid profile of the surveyed population by T2DM diagnosis status**

	T2DM categories				
	Diabetic	Prediabetic	Non-diabetic	Total	***p***value
	**n = 403**	**n = 207**	**n = 377**		
	Mean (SD)	Mean (SD)	Mean (SD)		
**Total cholesterol**	2.02 (0.45)	2.27 (0.47)	2.19 (0.49)	**987**	**<0.0001**
**Triglycerides**	1.87 (1.15)	1.63 (0.81)	1.49 (0.68)	**987**	**<0.0001**
**HDL-cholesterol**	0.48 (0.13)	0.54 (0.16)	0.53 (0.13)	**987**	**<0.0001**
**LDL-cholesterol**	1.35 (0.45)	1.26 (0.39)	1.19 (0.37)	**987**	**<0.0001**

*Abbreviation*: *T2DM* type 2 diabetes mellitus, *SD* Standard deviation.

**Table 6:** **Distribution of comorbid factors in the surveyed population by T2DM diagnosis status**

	T2DM categories				
	Diabetic	Prediabetic	Non-diabetic	Total	***p***value
	No. (%)	No. (%)	No. (%)		
**Hypertension**				**961**	**<0.0001**
Yes	226 (56.6)	97 (47.5)	139 (38.8)		
No	173 (43.4)	107 (52.5)	219 (61.2)		
**Hyperlipidemia**				**945**	**<0.0001**
Yes	222 (56.1)	99 (48.8)	133 (38.4)		
No	174 (43.9)	104 (51.2)	213 (61.6)		
**Obesity**				**946**	**<0.0001**
Yes	142 (36.0)	45 (22.2)	62 (17.8)		
No	252 (64.0)	158 (77.8)	287 (82.2)		
**Cardiac disease**				**952**	**0.08**
Yes	93 (23.4)	37 (18.2)	60 (17.1)		
No	305 (76.6)	166 (81.8)	291 (82.9)		

*Abbreviation*: *T2DM* Type 2 diabetes mellitus.

Triglycerides mean was significantly higher in patients with severe CAD compared to patients with mild CAD as well as healthy unaffected individuals (Table 2). Overall hyperlipidemia was associated with CAD (p < 0.0001). Low levels of HDL-C were associated with a severe CAD affection status (p = 0.02) (Table 2).

Both triglycerides and overall hyperlipidemia were significantly associated with the diabetes status in the 1330 stenosis free group (Table 3). Subjects with T2DM showed a trend towards a decrease in HDL-C levels although this did not reach the level of significance (Table 3). A strong correlation of other comorbid factors such as hypertension (p < 0.0001) and obesity (p < 0.0001) were highly associated with T2DM (Table 3).

**Figure 1:**
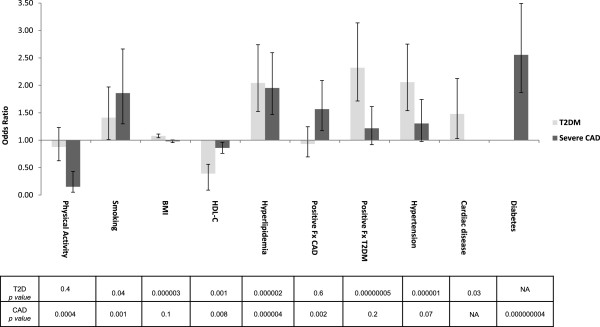
**Risk factors for type 2 diabetes mellitus and coronary artery disease.** Odds ratios and confidence intervals are shown for T2DM (light grey) and CAD (dark grey) respectively. For each risk factor, a *p* value shows significance for T2DM and CAD respectively. HDL-C was analyzed as a categorical variable with a threshold of ≥35 mg/dL for increased T2DM risk. A ratio of TC/HDL-C ≥4 increased risk of CAD. Abbreviations: T2DM, type 2 diabetes mellitus; CAD, coronary artery disease; BMI, body mass index; HDL-C, HDL-cholesterol; TGs, triglycerides; Fx, family history; NA, not applicable.

Studying reproductive health of women in the surveyed population showed a strong correlation between giving birth to a baby with a high weight and the occurrence of T2DM and prediabetes later in life (p < 0.0001) (Table 7). Retinopathy and peripheral neuropathy were significantly correlated with diabetes and prediabetes (p < 0.0001) (Table 8). We calculated the odds ratios of T2DM versus CAD in our T2DM (998 subjects) and CAD (991 subjects) groups. Figure 1 and Table 9 present an inferred risk of the major T2DM and CAD risk factors such as physical activity, smoking, BMI, HDL-C levels, hyperlipidemia, positive family history, and hypertension. The 1,330 stenosis free subjects replicated and confirmed the inferred risk of the factors found to increase T2DM in our surveyed population, such as BMI, triglycerides, overall hyperlipidemia, positive family history of T2DM, and hypertension. The protective role of physical activity against T2DM was also confirmed (Table 9).

**Table 7:** **Data on reproductive health of females in the surveyed T2DM and prediabetes populations**

	T2DM categories				
	Diabetic	Prediabetic	Non-diabetic	Total	***p***value
	No. (%)	No. (%)	No. (%)		
**Gestational diabetes**				**484**	**0.3**
Yes	9 (4.3)	2 (1.9)	3 (1.8)		
No	199 (95.7)	105 (98.1)	166 (98.2)		
**Baby weighing >4 kgs at birth**				**475**	**<0.0001**
Yes	75(37.3)	26 (23.9)	28 (17.0)		
No	126 (62.7)	83 (76.1)	137 (83.0)		
**Polycystic ovary syndrome**				**544**	**0.3**
Yes	9 (3.9)	6 (4.8)	14 (7.4)		
No	222 (96.1)	118 (95.2)	175 (92.6)		

*Abbreviation*: *T2DM* Type 2 diabetes mellitus.

**Table 8:** **Distribution of reported health complications in the surveyed population by T2DM diagnosis status**

	T2DM categories				
	Diabetic	Prediabetic	Non-diabetic	Total	***p***value
	No. (%)	No. (%)	No. (%)	**944**	**0.0008**
**Retinopathy**					
Yes	41 (10.4)	10 (5.0)	13 (3.7)		
No	354 (89.6)	192 (95.0)	334 (96.3)		
**Peripheral neuropathy**				**944**	**0.005**
Yes	61 (15.5)	25 (12.3)	27 (7.8)		
No	332 (84.5)	178 (87.7)	321 (92.2)		
**Liver disease**				**948**	**0.4**
Yes	18 (4.5)	5 (2.5)	11 (3.2)		
No	378 (95.5)	198 (97.5)	338 (96.8)		
Skin condition*				**948**	**0.6**
Yes	17 (4.3)	7 (3.4)	10 (2.9)		
No	380 (95.7)	196 (96.6)	338 (97.1)		

*Skin condition includes eleven psoriasis, six fungal infections, five dark spots on the skin, three vitiligo cases, three eczema, two lichen planus rashes, two back lipomas, and two itchy eruption of the skin. *Abbreviation*: *T2DM* Type 2 diabetes mellitus.

**Table 9:** **Unadjusted OR of association of selected risk factors**

	T2DM (n = 998)			Severe CAD (n = 991)			T2DM (n = 1,330)		
	OR	95% CI	***p***value	OR	95% CI	***p***value	OR	95% CI	***p***value
**Physical activity** (Ref: Inactive people)									
Active people	0.88	0.62-1.23	0.4	0.15	0.05-0.43	0.0004	0.46	0.24-0.88	0.02
Moderately active people	1.1	0.66-1.83	0.7	0.59	0.40-0.87	0.008	0.55	0.37-0.81	0.003
**Smoking** (Ref: Non-smokers)									
Current Smoker	1.41	1.01-1.97	0.04	1.86	1.30-2.66	0.001	0.55	0.40-0.75	0.0001
Ex-Smokers	1.4	0.96-2.02	0.08	1.23	0.73-2.08	0.4	0.79	0.55-1.13	0.2
**BMI** (Ref: < 30)									
≥ 30	1.08	1.04-1.11	<0.0001	0.98	0.95-1.01	0.1	2.29	1.74-3.02	<0.0001
**HDL-cholesterol** (Ref: Healthy)									
Yes	0.39	0.22-0.69	0.001	0.86	0.76-0.96	0.01	1.08	0.79-1.48	0.6
**Triglycerides** (Ref: Healthy)									
Yes	1.66	1.37-2.00	<0.0001	1	1.00-1.01	0.001	1.78	1.31-2.40	0.0002
**Hyperlipidemia** (Ref: Healthy)									
Yes	2.04	1.52-2.74	<0.0001	1.95	1.47-2.59	<0.0001	2.22	1.62-3.04	<0.0001
**Family history of CAD** (Ref: No family history of CAD)									
Yes	0.93	0.70-1.25	0.6	1.57	1.17-2.09	0.002	0.86	0.66-1.12	0.3
**Family history of T2DM** (Ref: No family history of T2DM)									
Yes	2.32	1.71-3.14	<0.0001	1.22	0.92-1.61	0.2	2.66	2.00-3.54	<0.0001
**Hypertension** (Ref: Healthy)									
Yes	2.06	1.54-2.75	<0.0001	1.3	0.98-1.74	0.07	3.08	2.30-4.12	<0.0001
**Coronary artery disease** (Ref: Healthy)									
Yes	1.48	1.03-2.12	0.03	NA	NA	NA	NA	NA	NA
**Type 2 diabetes mellitus** (Ref: Healthy)									
Yes	NA	NA	NA	2.56	1.87-3.49	<0.0001	NA	NA	NA

ORs are calculated among individuals having T2DM compared to non-diabetic and having severe CAD compared to no stenosis as outcome variables.

*Abbreviation*: *T2DM* Type 2 diabetes mellitus, *CAD* Coronary artery disease, *OR* Odds ratio, *BMI* Body mass index, *Ref* Reference.

Population enrollment shows significant association with T2DM diagnosis (OR = 1.41, 95% CI: 1.22 – 1.62, p-value = 1.73×10^-6^) where 42% of the case–control selected T2DM enrollees had a T2DM diagnosis, while 34% of the CAD study enrollees had T2DM. On the other hand, there is a significant inverse association of CAD diagnosis with enrollment in the T2DM study (OR = 0.26, 95% CI: 0.19 – 0.36, p-value < 2×10^-16^).

Results for variables such as hypertension, obesity and hyperlipidemia showed different levels of significant association with population enrollment in T2DM and CAD cohorts (Additional file 1).

Prediction of T2DM by metabolic syndrome variables, without or with inclusion of population for adjustment: hypertension (without: OR = 1.61, 95% CI: 1.42 – 1.83, p-value = 2.31 × 10^-13^; with: OR = 1.75, 95% CI: 1.54 – 2.00, p-value < 2 × 10^-16^), hyperlipidemia (without: OR = 1.53, 95% CI: 1.35 – 1.72, p-value = 3.33 × 10^-12^; with: OR = 1.52, 95% CI: 1.35 – 1.71, p-value = 7.63 × 10^-12^), triglycerides (without: OR = 1.49, 95% CI: 1.30 – 1.71, p-value = 7.82 × 10^-9^; with: OR = 1.48, 95% CI: 1.29 – 1.70, p-value = 2.08 × 10^-8^), BMI (without: OR = 1.17, 95% CI: 1.03 – 1.32, p-value = 0.0127; with: OR = 1.19, 95% CI: 1.05 – 1.35, p-value = 0.00515), and gender (without: OR = 1.06, 95% CI: 0.94 – 1.20, p-value = 0.322; with: OR = 0.94, 95% CI: 0.82 – 1.07, p-value = 0.332).

Prediction of CAD by metabolic syndrome variables computed in a single regression, without or with inclusion of population for adjustment: T2DM (without: OR = 1.85, 95% CI: 1.51 – 2.27, p-value = 2.45 × 10^-9^; with: OR = 1.97, 95% CI: 1.61 – 2.43, p-value = 9.46 × 10^-11^), hypertension (without: OR = 1.41, 95% CI: 1.17 – 1.70, p-value = 2.73 × 10^-4^; with: OR = 1.42, 95% CI: 1.17 – 1.71, p-value = 2.63 × 10^-4^), hyperlipidemia (without: OR = 1.52, 95% CI: 1.27 – 1.82, p-value = 4.98 × 10^-6^; with: OR = 1.56, 95% CI: 1.30 – 1.86, p-value = 1.49 × 10^-6^), triglycerides (without: OR = 1.08, 95% CI: 0.87 – 1.34, p-value = 0.471; with: OR = 1.09, 95% CI: 0.88 – 1.36, p-value = 0.414), BMI (without: OR = 0.87, 95% CI: 0.73 – 1.04 , p-value = 0.132; with: OR = 0.86, 95% CI: 0.72 – 1.03, p-value = 0.107), and gender (without: OR = 0.26, 95% CI: 0.22 – 1.31, p-value < 2 × 10^-16^; with: OR = 0.27, 95% CI: 0.22 – 0.32, p-value = <2 × 10^-16^).

## Discussion

Much about the pathology of T2DM and its etiology remains unknown. Researchers acknowledge that different genes and environmental factors may lead to disease processes that differ from person to person and from an environment to another. This study unravels some of the key environmental and behavioral factors that play a role in T2DM occurrence in the Lebanese population.

### Prevalence of T2DM and prediabetes

The small number of males in the survey sample (35.6%) was due to the fact that most men were at work at the time of the recruitment. In the randomly selected population, 51.7% of the participants were male compared to 48.3 females. Our surveyed population consisted of 40.8% diabetics, 20.7% of people with prediabetes, and 38% of unaffected healthy participants. The overestimation of affected people is probably due to the fact that campaigns were organized mainly with the help of endocrinologists for increasing T2DM awareness. Thus, people who knew they were affected and people with a positive family history had a higher interest in participating. This led us to investigate a population of 1,330 stenosis free participants from the FGENTCARD database. Although the prevalence of T2DM was not as high as in our surveyed population, it was 20.5% (Table 4). This confirms that Lebanon is facing an alarming increase of the prevalence of T2DM. This prevalence increased to 23.6% when we investigated control subjects that were above 55 years of age.

The prevalence of prediabetes was studied in the surveyed population only, as the information was not collected for the FGENTCARD participants. In the Lebanese population, the prevalence of prediabetes mirrors roughly the international tendency since approximately one in three U.S. citizens are known to have prediabetes [5]. Nearly 100% of the participants accurately self-reported T2DM and only 16% reported being aware of their pre-affection status (Table 10). These results indicate as expected that almost all individuals with T2DM were aware of their affection status, but alarmingly show that only one in six individuals had a prior knowledge of their prediabetes status. Awareness of prediabetes should be promoted on the national level as persons with prediabetes are at high risk for developing T2DM and 11% of persons with prediabetes who do not engage in moderate physical activity and do not lose weight will progress to T2DM [6].

**Table 10:** **Self-reported T2DM, prediabetes, and excess body weight in the surveyed population**

	Reported condition	Not reported condition	Total
	No. (%)	No. (%)	
**Self-reported T2DM**	405 (42.2)	555 (57.8)	**960**
**Self-reported prediabetes**	35 (4.1)	825 (95.9)	**860**
**Self-reported weight excess**	250 (26.3)	701 (73.7)	**951**

*Abbreviation*: *T2DM* Type 2 diabetes mellitus.

### Young onset of type 2 diabetes

A feature characterizing T2DM in the Lebanese population is the tendency to develop young onset diabetes. A multiethnic population-based cohort from Canada noted that the median age at diagnosis of diabetes was lower by 3 years in Chinese compared to Caucasians (55 years vs. 58 years) [7]. In our surveyed population the mean age of onset of T2DM was of 55 years and thus 3 years less than the median age at diagnosis in Caucasians, although it is still higher than the mean age of diagnosis of T2DM in East Asia that is typically around 50 years (Table 1) [8]. The young age at diagnosis may be due to an increasingly overweight population as well as the rise in caloric and fat intake in a region where exercise is not a defining part of the culture [9].

### Reproductive health of females and intrauterine environment

Once diagnosed with gestational diabetes mellitus, women are seven times more likely to develop T2DM during their lifetime compared with women without GDM history [10]. Because the information about GDM is largely missing in our cohort, we investigated the number of women who gave birth to a baby weighing more than 4 kilograms (9 pounds), since GDM and hyperglycemia in pregnancy have long been related to excessive fetal growth [11–14]. The link between birth weight and adult risk of T2DM has been observed in our cohort making it a general statement across different ethnicities. This is of particular concern since a comparison to offspring of normoglycemic mothers shows that offspring of mothers with GDM have increased adiposity at birth and increased risk of diabetes and obesity later in life [15, 16]. Given the potential transgenerational effects of maternal hyperglycemia, GDM screening, diagnosis and treatment should be routinely practiced in the Lebanese hospitals.

### Lifestyle environmental and comorbid risk factors

When participants from the surveyed population were asked about their weight, 26.3% admitted to be overweighed while 73.7% estimated not to have excess weight (Table 10). BMI measures showed that significantly more participants were overweight than what is self-reported with only 23.5% of the participants having normal weight, 37.1% and 30.1% being overweighed and obese respectively. BMI measures showed a significant association of overweight and obesity with diabetes. Physical activity was less frequent in diabetics compared to healthy participants but did not show statistically significant association with T2DM. The protective role of physical activity, as well as the association of a BMI ≥30 with T2DM were further confirmed in our stenosis free population underlining the increased risk of disease occurrence in the Lebanese population. These findings highlight the urgent need for timely interventions promoting physical activity and weight control nationwide since Lebanon shows high prevalence of overweight and obesity comparable with those observed in developed countries [17].

*Positive family history* of T2DM was one of the most significant factors predisposing to diabetes occurrence in our study population underscoring the need for screening in individuals with family history. One of the reasons to screen for diabetes is that it has an asymptomatic phase for which screening can be helpful in preventing major health problems in a sizable portion of the population [18].

*Hypertension* was positively associated with diabetes in the different cohorts. Since both hypertension and T2DM affect the same major target vascular tree, lowering blood pressure should be the primary goal in the management of the hypertensive diabetic patients. Comorbid factors such as *hyperlipidemia and* low *HDL-C levels* increased the risk of T2DM. T2DM is known to be associated with plasma lipid and lipoprotein abnormalities, including reduced HDL-C, and elevated triglycerides [19]. Prediabetics also exhibit an atherogenic pattern of risk factors that includes higher levels of TC, LDL-C, and triglycerides and lower levels of HDL-C [20, 21]. These plasma lipid and lipoprotein associated abnormalities contribute to the risk for atherosclerosis in the majority of patients with type 2 diabetes [22]. Although we reproduced the positive association of overall hyperlipidemia with T2DM in the stenosis free participants, HDL-C did not show a significant protective role, but rather a tendency towards lower levels in individuals with T2DM (Table 3).

In the surveyed sample, peripheral neuropathy and retinopathy were the two complications significantly associated with T2DM. This is expected since peripheral neuropathy, a common microvascular complication of diabetes [23], is often associated with concomitant retinopathy [24].

### T2DM versus CAD

There is a close relationship between T2DM and CAD since patients with T2DM have a two to four fold higher risk of a cardiovascular event with a faster progression when compared with non-diabetic patients [25]. Comparing our T2DM surveyed cohort to an age and sex-matched previously described Lebanese CAD cohort confirmed that the presence of cardiac disease increased the risk of T2DM by 1.48 fold, whereas T2DM increased the risk of severe CAD by 2.56 fold. Studying other known environmental risk factors confirmed that the risk of severe CAD is increased with sedentary life, smoking, decreased HDL-C level, hyperlipidemia, and positive CAD family history. Hence, there is a growing need to emphasize early and vigilant risk factor management in patients with T2DM and CAD to help reduce their morbidity and mortality.

Smoking correlates with hypertension in a generally surveyed population, including diabetics, but is anti-correlated among subjects that reached the stage of catheterization. Interactions of activity level and smoking variables and T2DM also show interactions between populations. This suggests that the course of progression and treatment leading to catheterization include behavior modifications impacting correlations between these populations.

Contrary to the behavioral variables, the prediction of both T2DM and CAD by metabolic syndrome variables appears to be unaffected by population, even though the proportions of these variables in the two populations are different.

While T2DM, as a part of the metabolic syndrome complex, is a strong predictor of CAD, the associations between behavioral contributions and disease are strongly impacted by progression of disease and treatment.

### Comparison with previous studies

A previous study aimed to determine the prevalence of diagnosed and undiagnosed diabetes in Greater Beirut in 2005 [26]. From a total of 3,000 exclusively Lebanese citizens with a mean age of 55.5, 11.3% were found to have previously diagnosed diabetes and 5.1% had undiagnosed diabetes. The combined prevalence of previously diagnosed and newly diagnosed diabetes was 15.8%. In the U.S., 6.3% of the population had diabetes: 4.5% diagnosed and 1.8% undiagnosed [27]. The ratio of known to unknown diabetes obtained in the Lebanese study was similar to the one recognized in the U.S at that time. Our results show that the prevalence of diagnosed diabetes in Lebanon had a sharp increase (2.6 fold in the surveyed population and 1.5 fold for the randomly selected population) among people ≥ 55 years old for both sexes. A comparable increase is seen in the U.S. where the prevalence of diagnosed diabetes increased 2.3 times from 1990 to 2010 [28]. This study provides evidence that there is a severe epidemic of diabetes including self-reported diagnosed and undiagnosed diabetes. These results underscore the urgent need for planning and delivering primary prevention to the Lebanese population.

## Conclusions

In conclusion, our study evaluates the number of prediabetics, diagnosed and undiagnosed T2DM in a group of the Lebanese participants. It shows a significant role for BMI, and positive family history in the predisposition to prediabetes and T2DM. It confirms comorbid factors such as hypertension, hyperlipidemia, and low levels of HDL-C to be associated with an increased risk of prediabetes and T2DM. It shows the effect of the *in utero* environment on the risk of T2DM later in life and gives an overview of the complications of T2DM in the Lebanese population. It compares well known risk factors in T2DM and CAD occurrence measuring odds ratios, showing the link between these two diseases that could be partly determined by an unhealthy lifestyle habits as overweight and physical inactivity. The results suggest the need to further educate the general public about the risk factors, complications and severity of T2DM and CAD prior to the development of disease. Observed associations between behaviors and disease depend heavily on progression of disease and treatment. Specifically, more advanced CAD and T2DM subjects enrolled through catheterization tend to have a negative association with smoking than those enrolled through advertisement in the T2DM cohort. The strongest correlations, regardless of enrollment, were between metabolic syndrome and T2DM.

## Electronic supplementary material

Additional file 1: **Collection of data and demographic characteristics.** A questionnaire gathered data on each subject’s demographic characteristics such as age, sex, marital status, origin, and parental consanguinity. Data on behavioral risks factors, smoking status, alcohol consumption and exercise pattern was also obtained. Results. Association of behavioral variables such as hypertension, smoking, obesity, and hyperlipidemia, with population enrollment. (PDF 79 KB)

## Abbreviations

T2DM: Type 2 diabetes mellitus
CAD: Coronary artery disease
IDF: International diabetes federation
MENA: Middle East and North Africa Region
GDM: Gestational diabetes mellitus
FGENTCARD: Functional genomic diagnostic tools for coronary artery disease
HbA1C: Glycated hemoglobin
FBS: Fasting blood sugar
BMI: Body mass index
TC: Total cholesterol
HDL-C: HDL-cholesterol
LDL-C: LDL-cholesterol
ADA: American diabetes association
SPSS: Statistical package for social sciences
OR: Odds ratio
SD: Size difference
Ref: Reference
NPRP: National priorities research program.
