# Supplementary material for: Multivariate epidemiologic analysis of type 2 diabetes mellitus risks in the Lebanese population
Source: Diabetol Metab Syndr. 2014 Aug 21;6:89. doi: 10.1186/1758-5996-6-89 (PMC4153892; doi:10.1186/1758-5996-6-89)
Supplement: Supplementary file 1 — Additional file 1: Collection of data and demographic characteristics. A questionnaire gathered data on each subject’s demographic characteristics such as age, sex, marital status, origin, and parental consanguinity. Data on behavioral risks factors, smoking status, alcohol consumption and exercise pattern was also obtained. Results. Association of behavioral variables such as hypertension, smoking, obesity, and hyperlipidemia, with population enrollment. (PDF 79 KB) [file 13098_2014_354_MOESM1_ESM.pdf]

Subjects and methods

Data Collection

A questionnaire gathered data on each subject’s demographic characteristics such as age, sex, marital status, origin, and parental consanguinity. Data on behavioral risks factors, smoking status, alcohol consumption and exercise pattern was also obtained. General health behavior comprised information about the number of times subject exercised more than 20 minutes per week. Subjects were classified into three categories: category 1: inactive people; category 2: people exercising once or twice per week; category 3: active people exercising three or more times per week. Information about smoking was collected and divided into three categories: category 1: non-smokers; category 2: ex-smokers; category 3: current smokers, with the number of cigarettes per day specified. Data on alcohol consumption categorized participants into non-drinkers, drinkers consuming less than seven drinks per week, and drinkers consuming more than seven drinks per week. The questionnaire inquired about the individual family history of T2DM, hyperlipidemia and cardiovascular disease. Participants were further asked for their medical history in terms of T2DM risk factors (obesity, hyperlipidemia, hypertension, and cardiovascular disease). In the surveyed population, the 998 participants were asked about prediabetes. For people who reported having diabetes, data on the diabetes type, the age of onset, and the management of diagnosed T2DM including regular checkups and changes in lifestyle since the diagnosis as well as self-reported T2DM complications such as retinopathy (disease of the retina that involves damage to the tiny blood vessels in the back of the eye), peripheral neuropathy (nerve damage throughout the body with symptoms such as pain, tingling, or numbness—loss of feeling—in the hands, arms, feet, and legs), liver disease (this includes nonalcoholic

fatty liver disease (NAFLD), cirrhosis, hepatocellular carcinoma, and acute liver failure), and skin problems (skin rashes that can occur in T2DM, collectively known as diabetic dermadromes) were obtained. All participants filled a detailed table for medication intake specifying the name, dosage, and duration of drugs intake. Female participants answered specific questions related to reproductive health such as history of Gestational diabetes and whether they delivered a baby with a birth weight ≥ 4 Kilograms.

Results

Results for behavioral variables shows a significant association with population enrollment (OR=5.59, 95%CI: 4.64 – 6.73, p-value<2x10-16), indicating T2DM enrollees appear to be more active than CAD enrollees. Similarly, T2DM enrollees are less often smokers than the CAD enrollees (OR=0.50, 95%CI: 0.42 – 0.58, p-value<2x10-16). This variation might be partly due to self-reporting bias due to how the question of smoking use was phrased.

Hypertension is strongly predicted by enrollment, with CAD enrollees being much more likely to have a diagnosis of hypertension (OR=0.42, 95%CI=0.37 – 0.48, p-value<2x10-16). In the pooled set, activity level appears to negatively associate with hypertension (OR=0.71, 95%CI: 0.60 – 0.85, p-value=2.03x10-4). However, there is a strong association between activity level and population. The association vanishes given adjustment with population, either with (OR=0.72, 95%CI: 0.41 – 1.28, p-value=0.26) or without (OR=0.93, 95%CI: 0.77 – 1.12, p-value=0.44) including the interaction. Hypertension negatively associates with smoking in the pooled set (OR=0.78, 95%CI: 0.70 – 0.87, p-value=9.56x10-6). That relationship persists under adjustment for population without interactions (OR=0.72, 95%CI: 0.64 – 0.80, p-

value=6.22x10-9) and with interactions (OR=0.29, 95%CI: 0.20 – 0.43, p-value=5.47x10-10). The negative association is present among CAD enrollees (OR=0.65, 95%CI: 0.58 – 0.73, p-value=9.39x10-13), which was observed previously, but is positive among the T2DM enrollees (OR=1.45, 95%CI: 1.07 – 1.98, p-value=0.018).

Obesity is anti-associated with T2DM enrollment compared to CAD (OR=0.78, 95%CI: 0.67 – 0.90, p-value=8.79x10-4). In the pooled set, activity level is anti-correlated with obesity (OR=0.62, 95%CI=0.51 – 0.76, p-value=3.45x10-6). Adjustment by population without interactions does not shift odds ratios (OR=0.66, 95%CI: 0.54 – 0.81, p-value=7.17x10-8), however inclusion of population interaction with activity renders the association insignificant (OR=0.80, 95%CI: 0.43 – 1.47, p-value=0.47). Among CAD enrollees, the anti-association of obesity with activity is significant (OR=0.69, 95%CI: 0.53 – 0.90, p-value=0.0059), and among T2DM enrollees, the anti-association is preserved (OR=0.61, 95%CI: 0.43 – 0.84, p-value=0.00342). Obesity is negatively related to smoking across all populations in all test configurations (data not shown).

Hyperlipidemia was not associated with population (OR=0.96, 95%CI: 0.84 – 1.10, p-value=0.568), activity level (OR=0.93, 95%CI: 0.78 – 1.11, p-value=0.443), or smoking (OR=1.00, 95%CI: 0.90 – 1.11, p-value=0.955).

T2DM is diminished by activity level (OR=0.81, 95%CI: 0.67 – 0.97, p-value=0.0268). Adjustment by population without (OR=0.70, 95%CI: 0.58 – 0.85, p-value=4.39x10-4) and with (OR=0.36, 95%CI: 0.19 – 0.66, p-value=0.00114) interactions, among CAD enrollees (OR=0.57, 95%CI: 0.43 – 0.74, p-value=5.553x10-5), but not among T2DM study enrollees (OR=0.91, 95%CI: 0.68 –

1.22, p-value=0.542). It is worth noting that the impact of the interaction between activity and population on T2DM was significant (OR=1.60, 95%CI: 1.07 – 2.40, p-value=0.0224). T2DM dependence on smoking shows significance in the CAD cohort (OR=0.76, 95%CI: 0.57 – 0.76, p-value=9.25□10-6). The negative association may reflect response to admonishment to quit smoking during the treatment period ultimately leading to catheterization. Among the T2DM cohort, the association is not significant (OR=1.26, 95%CI: 0.92 – 1.72, p-value=0.148). When interaction with population is included, the impact of smoking becomes significant (OR=0.46, 95%CI: 0.31 – 0.68, p-value=1.14□10-4). The population interaction term is strongly positive and significant (OR=1.65, 95%CI: 1.18 – 2.31, p-value=0.0032).
